# Influence of Model Structures on Predictors of Protein Stability Changes from Single-Point Mutations

**DOI:** 10.3390/genes14122228

**Published:** 2023-12-17

**Authors:** Cesare Rollo, Corrado Pancotti, Giovanni Birolo, Ivan Rossi, Tiziana Sanavia, Piero Fariselli

**Affiliations:** Department of Medical Sciences, University Torino, 10126 Torino, Italygiovanni.birolo@unito.it (G.B.); ivan.rossi@unito.it (I.R.); tiziana.sanavia@unito.it (T.S.); piero.fariselli@unito.it (P.F.)

**Keywords:** protein stability, single-point mutation, stability change, performance evaluation, machine learning

## Abstract

Missense variation in genomes can affect protein structure stability and, in turn, the cell physiology behavior. Predicting the impact of those variations is relevant, and the best-performing computational tools exploit the protein structure information. However, most of the current protein sequence variants are unresolved, and comparative or ab initio tools can provide a structure. Here, we evaluate the impact of model structures, compared to experimental structures, on the predictors of protein stability changes upon single-point mutations, where no significant changes are expected between the original and the mutated structures. We show that there are substantial differences among the computational tools. Methods that rely on coarse-grained representation are less sensitive to the underlying protein structures. In contrast, tools that exploit more detailed molecular representations are sensible to structures generated from comparative modeling, even on single-residue substitutions.

## 1. Introduction

Understanding the effect of variations on protein stability is critical in molecular biology and medical applications. Due to residue variations, protein stability influences protein functionality and its regulation within biological systems.

The effect of variations on protein stability is expressed by the difference in the Gibbs free energy of unfolding between a mutated protein and its wild-type ($\Delta \Delta G_{MW}=\Delta G_{M}-\Delta G_{W}$). The sign indicates whether the variation decreases (i.e., destabilizing variant) or increases (i.e., stabilizing variant) the protein stability.

Different studies [1,2,3,4] demonstrate that mutations can significantly hinder a protein’s ability to effectively bind and interact with key biomolecules such as DNA, RNA, and other proteins. Consequently, alterations in protein stability can initiate a chain reaction, thereby potentially leading to disease development. Therefore, predicting protein stability would enable the identification of disease-causing mutations, particularly in monogenic disorders where a single mutation can be pivotal [5,6,7,8]. In addition, disruptions in protein stability, especially those linked to pathogenic missense variants, are also known to contribute to functional loss in haploinsufficient genes.

Predicting the free energy of unfolding (ΔG) is an unsolved and highly complex problem, which is distinct from predicting the coordinates of protein sequences. The latter is generally considered to be a solved problem for proteins with high sequence identity to a known three-dimensional structure, which is achievable through comparative modeling [9,10,11].

Although it is more straightforward in theory, accurately predicting ΔΔG remains an open challenge despite its importance [12,13]. This is largely due to the intricate nature of protein structures and the complex interplay of forces that dictate protein stability. In recent years, various computational tools and predictors have been developed to estimate ΔΔG values from amino-acid sequence or protein structures, thereby employing different methodologies ranging from energy-based [14,15,16,17,18,19,20] to machine-learning approaches [21,22,23,24,25,26,27,28,29]. However, a major hurdle in this field is the frequent unavailability of experimental structures for both the wild-type and mutant forms of proteins. Consequently, homology-based tools like Modeller [30] and Rosetta [31] fill the structure–sequence gap.

However, a systematic evaluation of their impact on prediction tools is yet to be performed. Only recently, Pan and colleagues [32] systematically investigated the performance and robustness of ten widely used structural methods when presented with homology models built using templates at various sequence identity levels (ranging between 15% and 95%). They observed a decrease in performance for homology models built with templates having sequence identity below 40%, thereby suggesting a preference for sequence-based tools.

According to our current experimental validation, a single point mutation is a straightforward case for comparative modeling tools, where the final predicted structure should be very close to the original one, with just some small atom changes [9,10]. However, it is crucial to emphasize that constructing a protein structure and computing its free energy of folding are distinct challenges. Despite the significant strides made by Alphafold2 [33,34,35], the difficulty in accurately calculating free energies with atomistic simulations remains a well-known challenge in molecular modeling and drug design [36,37].

In this respect, we extend the work of Pan et al. [32] by evaluating cases of extremely high sequence identity (just a single residue substitution) and exploring the effect of some structure-based methods when provided with homology-based protein models.

The present study focuses on a comparative analysis of several ΔΔG predictors, namely ACDC-NN, MAESTRO, DDGun, FoldX, Korpm, DDMut, and PoPMuSiC, which are designed to estimate the impact of single amino-acid variations on protein stability. Our analysis compares the performance of these predictors using both experimentally determined and computationally modeled protein structures, thereby focusing on those generated by Modeller and Rosetta.

## 2. Materials and Methods

For the comparison of the mentioned methods, we used the Ssym dataset [38], which is composed of 684 protein variants (342 direct and 342 reverse), with 19 experimental structures related to the direct variants and 342 experimental structures for each of the reverse variants. All 3D structures were solved using X-ray crystallography, with a median experimental resolution of 1.8Å. The availability of an even distribution of direct and reverse variants, with the respective experimental structure, is a unique and crucial property of the Ssym dataset, since it allows for the assessment of the ΔΔG predictors with respect to the required antisymmetry property ($\Delta \Delta G_{MW}=-\Delta \Delta G_{WM}$).

### 2.1. ΔΔG Predictors

A total of seven protein stability predictors were tested. Specifically, these included the following:

**ACDC-NN**: This is a neural network-based method that inherently satisfies antisymmetry properties. It processes local amino-acid information surrounding the mutation site, thereby incorporating multiple sequence alignments and considering the two amino acids involved in the mutation [29];

**DDGun3D**: This untrained method merges evolutionary information with statistical potentials to predict ΔΔG. DDGun3D, unlike the sequence-based DDGun, integrates the structural information evaluated by the Bastolla–Vendruscolo statistical potential and adjusts the weights based on the accessibility of the mutated amino acid. It also incorporates antisymmetric features and supports extensions for multiple variant predictions [39];

**DDMut**: This is a deep learning-based tool that uses a Siamese neural network for both forward and hypothetical reverse mutations, thus addressing antisymmetry. The network also integrates optimized graph-based signatures with physicochemical properties, thereby enhancing its predictive accuracy [22];

**MAESTRO**: This predictor utilizes a hybrid approach through an ensemble of neural networks, support vectors, and multiple linear regressors in a multiagent prediction method based on statistical scoring functions (SSFs) [17];

**FoldX**: This predictor employs an empirical force-field approach, thereby predicting single-point variation effects via a linear combination of empirical free energy terms, including contributions from entropy, Van der Waals forces, hydrogen bonds, and electrostatic interactions [16];

**PoPMuSiC**: This is an energy function-based method providing a linear combination of 13 statistical potentials, two volume-dependent terms of the wild-type and mutant amino acids, and an independent term. The coefficients depend on the solvent accessibility of the mutated residue, which is based on a sigmoid function whose parameters are optimized through a neural network [18];

**Korpm**: This predictor utilizes a simple residue-based orientational potential, thereby focusing on three backbone atoms of amino acids. This theoretical approach to stability prediction requires the parameterization of 12 amino acid-dependent weights. The method is designed to be almost antisymmetric, thus performing similarly for both direct and reverse mutations. It simplifies the complexity of protein stability prediction by using a straightforward sum of energy terms, thereby aiming at reducing the susceptibility to overfitting [19].

### 2.2. Protein 3D-Structure Models

**Modeller**: Modeller specializes in the comparative modeling of protein three-dimensional structures using known protein structures as templates. It builds the tertiary structure of a protein sequence primarily based on homology, thus aligning it to one or to multiple template structures. The algorithm generates an initial model and refines it iteratively, thus optimizing the spatial arrangement of the atoms. Modeller is particularly useful for modeling proteins when there are closely related structures available, and its speed and simplicity are especially advantageous for large-scale simulations [30].

**Rosetta**: Rosetta is a versatile suite for protein structure prediction and design. It uses both template-based and ab initio methods; the latter critical for proteins without close homologous structures [31]. When using the RosettaCM method [40], comparative models are built either from the structures detected and aligned by HHSEARCH, SPARKS, and Raptor or from a specific and provided template structure. In the first case, loop regions are assembled from fragments and optimized to fit the aligned template structures. It is worth mentioning that the whole procedure can be computationally demanding, especially for large proteins or in the absence of homologous structures, thus possibly requiring significant resources and time.

We used the Modeller version 9.24, setting max_iterations=900, min_atom_shift=0.001, and md_time_step=4.0. For Rosetta, we employed the Robetta server with the default parameters by using the comparative modeling (CM) method [40].

### 2.3. Assessment of Predictors’ Performance

We quantified the correlation between actual and predicted ΔΔG values using Pearson correlation coefficients (denoted as *r*), while the accuracy of the predictions was calculated in terms of root mean square error (RMSE) and mean absolute error (MAE). To evaluate the antisymmetry of ΔΔG predictors, we considered the Pearson correlation coefficient for direct and reverse mutations ($r_{d-r}$), which is defined as

$$r_{d-r}=\frac{\mathrm{Cov}\left(\Delta \Delta G^{dir},\Delta \Delta G^{rev}\right)}{\sigma_{dir}\sigma_{rev}},$$

where Cov represents the covariance between the direct and reverse ΔΔG predictions, and σ is the standard deviation. Considering that many predictors are trained on datasets with a preponderance of destabilizing mutations, we utilized the bias score (〈δ〉) to gauge the tendency towards a particular variant class:

$$\langle \delta \rangle =\frac{\sum_{i=1}^{N}\left(\Delta \Delta G_{i}^{\mathrm{dir}\;}+\Delta \Delta G_{i}^{\mathrm{rev}\;}\right)}{2N}.$$

In an ideal scenario, a predictor that shows perfect antisymmetry and no bias would have an $r_{d-r}$ value of −1 and a 〈δ〉 of 0.

## 3. Results

**Figure 1:**
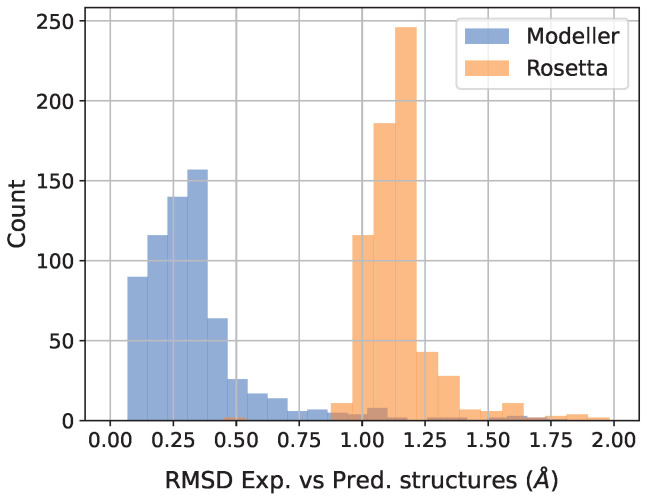
Distribution of RMSD values between residues’ distance matrices of experimental and Modeller/Rosetta predicted protein 3D structures.

We compared the three-dimensional protein structures generated by Modeller and Rosetta and their experimentally determined counterparts. This comparison was performed by calculating the root-mean-square deviation (RMSD) between the residue distance matrices of the experimental and the modeled structures in all protein variants. To better capture side-chain variations, we calculated the distances between residue pairs by considering the minimum separation among all pairs of atoms of each residue rather than the positions of the α carbon alone. The median RMSD values for the Modeller and Rosetta structures were 0.30Å and 1.14Å, respectively. The obtained distributions are shown in Figure 1. Differences in the 3D structures with respect to the experimental ones were within the experimental resolution of 1.8Å, thereby suggesting that the predicted geometries are accurate. However, it is crucial to note that this geometric accuracy does not inherently confirm the stability of the modeled energetic profile, which could be a key factor in the effectiveness of the ΔΔG predictors.

The ΔΔG prediction tool was assessed individually for its performance on direct, reverse, and combined (total) sets of mutations.

However, benchmarking the methods is not the purpose of this paper, and the presented results are not intended to compare their performance outcomes. The Ssym dataset does not represent a blind test set, since the proteins in this dataset share significant similarities with those used in the training or calibration of the ΔΔG predictors.

**Figure 2:**
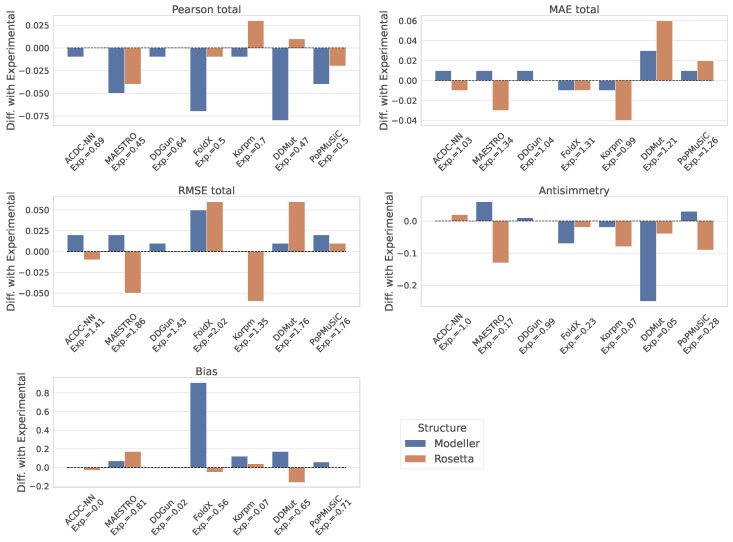
Comparative analysis of ΔΔG predictors’ performance (on the total set of mutations) in terms of Pearson correlation, MAE, RMSE, antisymmetry, and bias. Differential scores between experimental and Modeller/Rosetta-derived structures are displayed for each metrics. The names of the models and their respective absolute metric values on the experimental structures are annotated on the x axis.

The outcomes measured using experimental protein structures served as a benchmark for evaluating the corresponding metrics obtained from the predicted structures according to Modeller and Rosetta. Table 1 specifically reports these performance metrics for the Ssym experimental structures. To visualize the discrepancies in performance across experimental, Modeller-, and Rosetta-derived structures, refer to Figure 2.

In analyzing the performance variations of the ΔΔG predictors as elucidated by the bar plots, the presence of a heterogeneous response among the models is evident when utilizing nonexperimental structures for ΔΔG prediction. This is particularly highlighted by the differences in the Pearson correlation coefficients, which can be a proxy for the robustness of each model in the face of structural data derived from computational predictions. In particular, when using the Modeller structures, the performance deteriorations for models like FoldX, DDMut, and PoPMuSiC were significantly worse with respect to those caused by the Rosetta structures. On the other hand, ACDC-NN and DDGun predictors were the most robust ones according to all the considered metrics, thus showing only slight performance changes when using either the Modeller or Rosetta structures. Notably, in some instances, the utilization of Rosetta-derived structures appeared to have a favorable effect on the model performance. Specifically, models such as MAESTRO and Korpm showed not only a decreased deviation in the MAE and RMSE when using Rosetta structures compared to the experimental data, but also, in the case of Korpm, an improvement in the Pearson correlation coefficient was exhibited. This suggests that the advanced modeling capabilities of Rosetta, perhaps in capturing certain structural features or accommodating the inherent variability in protein folding, could be more closely related to the underlying algorithms of these particular predictors.

**Figure 3:**
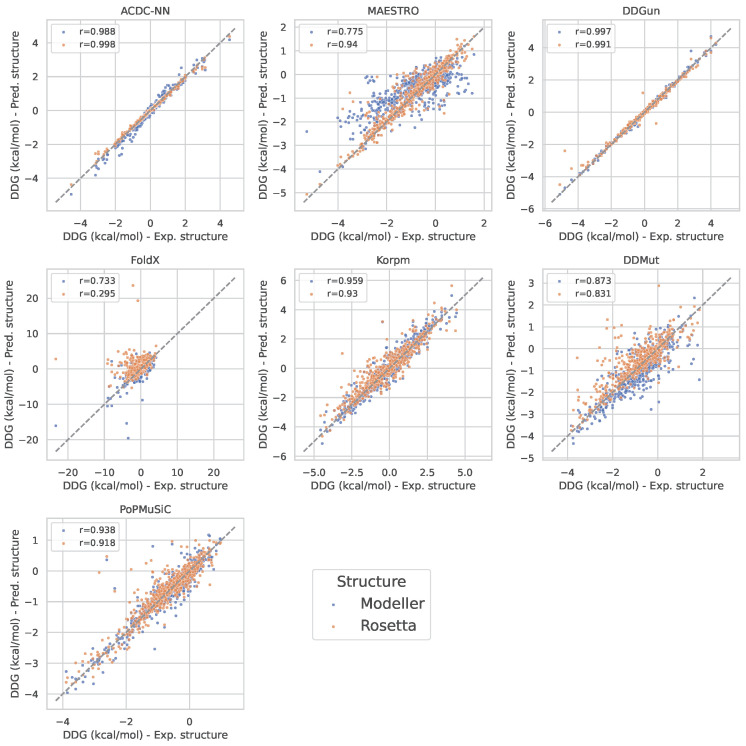
ΔΔG values, expressed in kcal/mol, obtained from experimental and from Modeller/Rosetta-predicted structures, for all the models. Pearson correlation coefficients *r* are annotated in each legend.

In order to further quantify the agreement of the predicted ΔΔG values between the experimental structures and the Modeller/Rosetta structures, we computed the Pearson correlation between the predictions for all the models (Figure 3). This analysis agrees with the previous differential metric evaluations, thereby reinforcing the observation of heterogeneity in the model robustness. Specifically, the ACDC-NN and DDGun exemplified resilience, thereby displaying Pearson coefficients that indicate strong agreement between predictions based on the experimental and computational structures. This robustness suggests that these models are well tuned to accommodate variations in input structural data, thereby making them reliable when the experimental structures are not available. In contrast, certain models, such as FoldX, exhibited a discernible decrease in the Pearson correlation, particularly with Modeller-derived structures. This points to a potential reliance on the high-fidelity structural data that computational models may not always provide, which could affect the utility of such predictors in scenarios where only predicted 3D structures are accessible. Furthermore, for all the considered models (with the exception of MAESTRO), the agreement between the ΔΔG predictions from Rosetta and the experimental structures was either comparable (ACDC-NN and DDGun) or higher (FoldX, Kropm, DDMut, and PoPMuSiC) than the one with Modeller.

### Comparative Analysis Based on Solvent Accessibility

**Figure 4:**
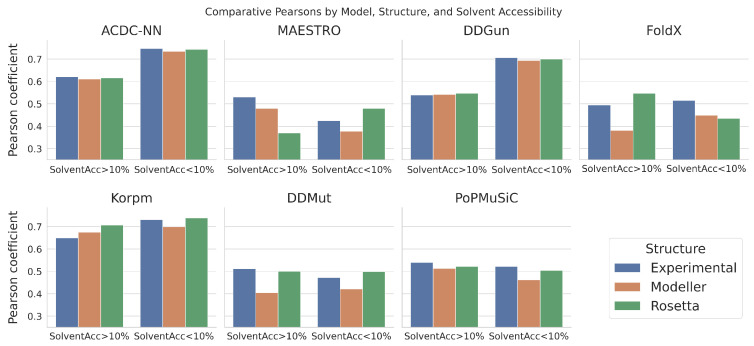
Pearson coefficients comparison between ΔΔG predictions from experimental and Modeller/Rosetta structures, which are categorized according to the solvent accessibility of the mutated residue.

Solvent accessibility is a critical parameter in assessing protein stability changes after single amino-acid mutations. It quantifies the exposure of residues to solvent molecules and significantly influences the energetic consequences of mutations. Residues with high solvent exposure often contribute to a protein’s interaction with its environment, while those with low solvent accessibility are typically involved in maintaining the protein’s internal structure. For the purposes of this study, we evaluated how and if the use of experimental or predicted protein structures affects the performance of ΔΔG predictors differently, wherein we focused separately on mutated amino acids with high or low solvent accessibility (greater or lower than 10%). The results obtained are shown in Figure 4 in terms of the Pearson correlation for all models.

In line with what was observed in the previous analysis, it is also possible to outline how the predictors behave differently when using experimental or predicted structures. In particular, the ACDC-NN and DDGun predictions proved to be robust for both high and low solvent accessibility categories. Notably, for both predictors, the performance outcomes for the low solvent accessibility category were significantly higher than those for the high solvent accessibility group. On the other hand, the MAESTRO and FoldX models were the most heterogeneously affected by the use of the Modeller or Rosetta structures, thereby showing discordant and incongruous behaviors for the two accessibility groups.

## 4. Discussion

The differential performance of ΔΔG prediction models, as evidenced by our study, underscores the nuanced interplay between protein structural data sources and the predictive accuracy of computational tools.

The comparative analysis of ΔΔG predictors highlights that the impact of using Modeller or Rosetta for structural predictions varies significantly between different models. For a subset of ΔΔG predictors, specifically the ACDC-NN and DDGun, the performance remained consistent, irrespective of the structural source. This was confirmed by all the metrics of interest and also when stratifying the performance with respect to the solvent accessibility of the mutated residue.

This finding is particularly noteworthy, thereby suggesting that these models possess inherent robustness and are less sensitive to the nuances of the input structure, be it experimentally derived or computationally predicted. In contrast, other models displayed a substantial performance disparity when using predicted instead of experimental structures, as well as distinct variations when choosing between Modeller or Rosetta, with Modeller leading to a greater performance deterioration (especially with the FoldX, Korpm, and DDmut models, as shown by the Pearson correlation metrics presented in Figure 2). This divergence indicates a dependence on the local atomic variations of some predictors to maintain stable performance.

The pronounced difference in performance with computationally modeled structures, which was particularly evident in purely energy-based models like FoldX, may stem from their heightened sensitivity to subtle deviations in predicted versus experimental structures. These models, which do not incorporate machine-learning techniques, heavily rely on the accuracy of the structural input to interpret the protein’s energy landscape, thereby making them more vulnerable to minor discrepancies in computationally derived structures.

Finally, the practical implications of these results are significant. Given that Modeller is less computationally intensive and faster than Rosetta, it presents a valuable tool for scenarios requiring a rapid model generation. Our analysis indicates that, with the correct model choice, the use of Modeller-derived structures need not be a compromise in accuracy, thus offering a computationally efficient alternative to the more resource-intensive Rosetta and to experimental structure determination methods.

## 5. Conclusions

Our study extends the insights gained from the recent investigation by Pan et al. [32], which demonstrated a decrease in the performance of structure-based tools for homology models built using templates with low sequence identity. Our research further explored this domain, thereby focusing on cases with very high sequence identity that were limited to single-residue substitutions. In this range, comparative modeling achieved impressive precision, thus generating structures that were indistinguishable from experimental ones. The natural expectation is that those comparative-based model structures would not have impacted the results of the computational tools predicting the free energy changes.

However, when we evaluated the effects of the structural inputs from Modeller and Rosetta on various ΔΔG predictors, we uncovered significant variations in their performance. As expected, predictors that exploit a coarse-grained representation of the protein structures showed less sensitivity to the source of the protein structure. On the contrary, tools relying on detailed molecular representations, such as FoldX, were more influenced by the slight variation of the atomic structures, although the predicted structure was derived through a single-point mutation from a known experimental model.

Our findings underscore the importance of careful model selection in protein stability analysis, especially when experimental structures are unavailable. A key takeaway is the potential utility of Modeller as a time-efficient and computationally frugal option, without necessarily compromising the accuracy of predictions, thereby providing the choice of a ΔΔG predictor that is robust with respect to slight structure variations. This aspect is of substantial relevance in research scenarios where the rapid generation of protein models is a priority.

## Figures and Tables

**Table 1:** ΔΔG prediction performances of different models on the 684 protein variants of the Ssym dataset when using the experimental 3D protein structures. Pearson correlation *r*, MAE and RMSE metrics have been separately computed on the sets of direct mutations (*dir.*), reverse mutations (*rev.*), and on the total dataset (*total*).

	$r_{\mathrm{total}}$	$r_{\mathrm{dir}.}$	$r_{\mathrm{rev}.}$	${\mathrm{MAE}}_{\mathrm{total}}$	${\mathrm{MAE}}_{\mathrm{dir}.}$	${\mathrm{MAE}}_{\mathrm{rev}.}$	${\mathrm{RMSE}}_{\mathrm{total}}$	${\mathrm{RMSE}}_{\mathrm{dir}.}$	${\mathrm{RMSE}}_{\mathrm{rev}.}$	$r_{d-r}$	〈δ〉
ACDC-NN	0.69	0.61	0.61	1.03	1.03	1.03	1.41	1.41	1.41	−1.00	0.00
MAESTRO	0.45	0.59	0.22	1.34	0.90	1.78	1.86	1.27	2.30	−0.17	−0.81
DDGun	0.64	0.55	0.53	1.04	1.02	1.05	1.43	1.41	1.45	−0.99	−0.02
FoldX	0.50	0.57	0.38	1.31	1.16	1.47	2.02	1.89	2.14	−0.23	−0.56
Korpm	0.70	0.57	0.49	0.99	0.96	1.02	1.35	1.30	1.39	−0.87	−0.07
DDMut	0.47	0.81	−0.02	1.21	0.67	1.76	1.76	0.94	2.30	0.05	−0.65
PoPMuSiC	0.50	0.63	0.25	1.26	0.86	1.66	1.76	1.21	2.18	−0.28	−0.71

## Data Availability

Experimental protein structures of the Ssym dataset are available at the protddg-bench repository https://protddg-bench.github.io/ssym/, accessed on 24 November 2023.
